# Investigation of Degradation of Composites Based on Unsaturated Polyester Resin and Vinyl Ester Resin

**DOI:** 10.3390/ma15041286

**Published:** 2022-02-09

**Authors:** Przemysław Pączkowski, Andrzej Puszka, Barbara Gawdzik

**Affiliations:** Department of Polymer Chemistry, Faculty of Chemistry, Institute of Chemical Sciences, Maria Curie-Sklodowska University in Lublin, Gliniana 33, 20-614 Lublin, Poland; przemyslaw.paczkowski@mail.umcs.pl (P.P.); andrzej.puszka@mail.umcs.pl (A.P.)

**Keywords:** lignin, biofiller, composites, unsaturated polyester resin, vinyl ester resin, degradation study, immersion test, chemical resistance, accelerated aging test, microwave irradiation

## Abstract

This study compares the degradation process of unsaturated polyester resin (UPR) and vinyl ester resin (VER) and their biocomposites with kraft lignin. In order to study their degradation, accelerated aging, immersion in different solvents, microwave radiation and high temperature were applied. The results show that, depending on the conditions, the degradation assumes a different course. The VER resin is more chemically resistant than the UPR resin. In the case of the composites immersed in an aggressive solvent (acetone), it can be observed that the polymer matrix is degraded, whereas in water only a small increase of weight takes place. Immersion in NaOH initiates the degradation process consisting in the hydrolysis of ester bonds, which are especially observed for pure resins. Under the influence of UV radiation and microwaves, the resins are additionally cross-linked. Thermogravimetric analysis shows that in the case of composites heated to 1000 °C, a residual mass remains, which is carbonized with lignin. In turn, composites treated with microwaves lost weight.

## 1. Introduction

Lignin is a natural biopolymer containing aromatic moieties in its chemical structure. It is mainly applied in the paper industry, but it can also be a unique precursor for the production of activated carbons, adhesives, biofuels, carbon fibers, and as a source of fine chemicals and phenolic monomers. Most of the lignin from the pulp and paper industry is used for energy production and is burned in a chemical recovery stage.

There are three phenolic monomers in the chemical structure of lignin: coumaryl, coniferyl and syringyl. These molecules are collectively called monolignols [1]. The structure of technical lignins vary substantially from native lignin based on the extraction process used (organosolv, sulfite, sulfate alkaline) due to the harsh conditions involved, including the addition of various chemicals and high temperatures. Kraft lignin is a type of industrial lignin whose world production exceeds 85% of the total lignin production [2].

Lignin possesses antioxidative, antimicrobial, and UV-blocking(-shielding) proper-ties due to its abundance of radical-scavenging phenolic and methoxyl groups [3,4,5]. Its properties are exploited in the production of new biomaterials such as biodegradable packaging, nanocomposite membranes, self-healing materials for biomedical and tissue engineering, and composite extracted from municipal solid waste [6,7,8,9,10,11,12,13,14,15].

Unsaturated polyester resins (UPR) are an important class of high-performance engineering polymers used in a variety of applications, mainly in molding such as compression, injection or resin transfer, pultrusion, filament winding and hand lay-up process [16]. A total of 85% of the reinforced polymer products such as boat, car and airplane parts and chairs were manufactured with them [17]. The global market of the unsaturated polyester resins will grow at a constant 5.3% Compound Annual Growth Rate (CAGR) over the forecast period (2019–2029) [18].

Unsaturated polyester resins are obtained by dissolving the starting unsaturated polyesters in vinyl, allyl or acrylic monomers. In industry, styrene is the most frequently used cross-linking monomer. The tendency to diminish styrene content reduces the harmful effects of the exposure of workers to the poisonous gas, thus raising the safety standards in the many industries. Styrene vapors can cause memory loss in workers, concentration difficulties, brain and liver damage, and even cancer [19]. Unsaturated polyester resins with reduced styrene content and slight shrinkage, are a green alternative [20].

Vinyl ester resins (VER) are a specific type of unsaturated polyester resin used where increased chemical resistance and strength are required. These materials are formed by the reaction of an epoxy resin with methacrylic acid. The resulting epoxy acrylates are dissolved in monomers. The obtained liquid resins can be used in the same manner as UPRs [21]. After cross-linking, the obtained materials are characterized by greater resistance to degradation, better impact and mechanical properties as well as better resistance to chemicals, with higher production costs. The formed network of vinyl ester resins creates stronger bonds compared to those of UPRs. The vinyl ester resins can be treated as a compromise between the unsaturated polyester resins and the epoxy resins [22].

Vinyl esters show better chemical and hydrolytic stabilities compared to the UPR because the unsaturated C=C double bonds placed at the end of the molecular chain are readily available during the cross-linking process and therefore practically complete conversion takes place after curing. Vinyl ester is characterized by having smaller number of open sites in the chain [23], which makes it much more resistant to the hydrolysis during water penetration and the formation of osmotic bubbles within it. Their chemical structure causes them to shrink less during curing which is favorable for removing the laminates from the molds. The VERs are more tensile tolerant than polyesters, which is what makes them more capable of absorbing impacts without being damaged [23]. They are also less prone to cracking caused by stress.

Generally, both UPR and VER are used interchangeably depending on the application [22]. Their composites are treated as extremely stable because they are infusible and insoluble materials due to their crosslinked network structure. At the end of their lifecycle, such materials become a crucial issue in terms of disposal. Most of these materials are landfilled or incinerated without any attempt to recycle [24]. The problem would be less burdensome if effective conditions for the process of their degradation were found.

Few attempts were made for the UPR composites using glass fiber fillers. Natural fillers such as wood flour or sawdust, maize cob, peanut shell, jute, bamboo, ramie and kenaf fiber are more and more popular and are used today [25,26,27,28,29,30,31,32,33]. For the VER, incorporation of kenaf, coconut or date palm seed are reported [34,35,36,37].

That makes sense to investigate the degradation conditions for such composites.

It is known that oxygen in the air, high temperature, hydrolysis associated with a humid atmosphere, light with a wavelength (>300 nm), biological attack, mechanical stress, contact with aggressive liquids, high-energy radiation and some living organisms have a possible detrimental effect on polymers [38,39,40,41,42,43,44,45,46]. Therefore, it makes sense to investigate the degradation conditions for such composites.

Our previous study showed that not only the composites of unsaturated polyester resins with biofillers but also the pure crosslinked resin can be colonized by some bacteria [47]. Settlement of bacteria is easier for the composites after aging when their original structure is slightly degraded.

This paper presents the results of research on the degradation of unsaturated polyester resin and vinyl ester resin composites containing the common biofiller—lignin. Degradation was in the presence of solar radiation (accelerated aging chamber), solvents of different nature (immersion test), microwaves (microwave reactor) and under the influence of high temperature (thermogravimetric analysis).

## 2. Materials and Methods

### 2.1. Chemicals

The mixture of unsaturated orthophthalic resin based on the recycled PET with styrene, Estromal 14PB-06 NZ (non-volatile content 61.2 wt.%, viscosity at 23 °C 356 mPas, acidic value 13.4 mg KOH g^−1^, and reactivity factor 1.53) and the vinyl ester/styrene resin, EBE1 (non-volatile content 30.57 wt.%, Brookfield viscosity at 25 °C 1520 cP, acidic value 7.6 mg KOH g^−1^, and water content 0.06%) were provided by LERG (Pustków, Poland). Methyl ethyl ketone peroxide (MEKP, Luperox DHD-9) as an initiator was provided by Sigma-Aldrich (St. Louis, MO, USA). A 4% solution of polymeric cobalt as an accelerator was synthesized in the Department of Polymer Chemistry, Institute of Chemical Sciences, Maria Curie-Sklodowska University in Lublin (Lublin, Poland). *N*,*N*′-Diethylaniline (DEA) as a co-accelerator was provided by Fluka Chemie AG (Buchs, Switzerland), whereas kraft lignin, alkali with a small sulfonate content was bought from Sigma-Aldrich (St. Louis, MO, USA).

### 2.2. Curing Conditions

The composites were prepared mixing the UPR or VER with 10 weight percentages of kraft lignin.

For curing of the UPR and its composite, 1.1 wt.% MEKP as an initiator and 0.25 wt.% of 4% polymeric cobalt solution were used. The VER requires less amount of MEKP (1.0 wt.%) and cobalt accelerator (0.12 wt.%) in favor of using DEA as a co-accelerator (0.06 wt.%). The same amounts of initiator and accelerators were used for the composites.

The prepared mixtures were mixed well until homogeneous and then poured into the cuboid-shaped molds. They cured at room temperature for 24 h and then at 80 °C for 10 h for post curing [48].

In Table 1 the compositions of the prepared materials are presented.

### 2.3. Specimen Preparation

In order to investigate the properties of the UPR and VER composites, their specimens were prepared in an appropriate manner. The cuboid composite samples 65 mm × 10 mm × 4 mm were cut with the MFG 8037P CNC milling machine from Ergwind (Gdańsk, Poland). The specimens were immersed in different solvents or placed in an accelerated aging chamber or microwave reactor. A reference sample that was not used in the degradation studies was applied.

### 2.4. Research Methods

#### 2.4.1. Immersion Degradation

The behavior of the cured UPR and VER resins as well as their composites with 10% of lignin in the presence of chemical aggressive liquids was determined according to the EN ISO 175: 2010 standard [49]. The cuboid specimens were immersed separately in the airtight containers with 50 mL of the tested liquid. Then they were placed at room temperature (23 °C ± 2 °C) in the dark. The chemical resistance of the samples was studied in acetone, toluene, distilled water, 10% HCl, 2% Na_2_CO_3_ and 1% NaOH. Periodically, the specimens were removed from the solvents, rinsed with distilled water and gently wiped.

The mass change (Δ*m*) was determined using Equation (1) [49]:

(1) $$\Delta m=\frac{m_{i}-m_{0}}{m_{0}}\times 100$$

where

*m*_0_ is the initial specimen mass;

*m_i_* is the specimen mass after the immersion test.

#### 2.4.2. Accelerated Aging Test

The accelerated aging test was performed using a Xenon Arc Lamp simulator Atlas Xenotest Alpha+ (Chicago, IL, USA). The irradiation source of this apparatus consists of a centrally located xenon arc lamp in the test chamber emitting radiation similar to natural sunlight with an irradiance of 60 W m^−2^. A chamber temperature of 38 °C, a black standard temperature of 65 °C, a relative humidity of 50%, and a daylight filter system were selected. A continuously dry exposure period of 400 h was used as the environmental conditions and degradation times for the accelerated aging process. The abovementioned parameters mimic the typical atmospheric conditions on which materials might be exposed. The test procedure was followed according to the following EN ISO 4892-2:2013 standard [50]. Each sample was exposed to a dosage (NTM) about 72,000 kJ m^−2^.

#### 2.4.3. Microwave-Assisted Degradation

The microwave irradiation was applied using a MAS-II Plus Microwave Synthesis Workstation from SINEO Microwave Chemistry Technology Co., Ltd. (Shanghai, China). The samples were exposed to microwave irradiation with a power of 1000 W until the temperature reached 150 °C for 20 min. The procedure was repeated several times, and each time the change of mass (Δ*m*) was determined according to Equation (1).

#### 2.4.4. Thermo-Oxidative and Thermomechanical Degradation

Thermal and thermomechanical properties were determined using a thermal analyzer and a dynamic mechanical analyzer.

The thermal stabilities of the UPR- and VER-based composites were evaluated by the thermogravimetric (TG, DTG) analysis. The TG scans were collected by means a Netzsch Simultaneous Thermal Analyzer STA 449F5 Jupiter (Selb, Germany) from 30 to 1000 °C at the heating rate of 10 °C min^−1^ in the oxidative atmosphere (air). The test procedure was in accordance with the EN ISO 11358-1:2014 standard [51].

The dynamic mechanical analyzer (DMA) Q800 from TA Instruments (New Castle, DE, USA) equipped with a dual-cantilever device was used to determine the thermomechanical properties of the UPR- and VER-based composites. The temperature scanning from −50 °C to 200 °C with a constant heating rate of 3 °C min^−1^ at a sinusoidal distortion of 10 µm amplitude and 1 Hz frequency was made. The specimens (65 mm × 10 mm × 4 mm) were studied before and after the immersion in liquid chemicals, UV radiation and microwaves. The test procedure was in accordance with the EN ISO 6721-1:2019 standard [52]. The result was only single measurement before and after degradation. The values of storage modulus (*E’*), glass-transition temperature (*T_g_*), mechanical loss factor (*tan δ*), and Full Width at Half Maximum (*FWHM*) were determined.

#### 2.4.5. Mechanical Properties

Mechanical properties were determined using a mechanical testing machine and a hardness tester.

The mechanical properties of the UPR and VER based samples were determined on the ZwickRoell Z010 mechanical testing machine from ZWICK GmbH Co (Ulm, Germany). Determination of mechanical properties based on the three-point bending test, where the samples of 65 mm × 10 mm × 4 mm diameter were used with a span of 50 mm between the supports. The bending speed was 5 mm min^−1^. The measurements were made according to the EN ISO 178:2019 standard [53]. Finally, the arithmetic averaging of five measurements was taken for UPR and VER samples. The flexural modulus (*E_mod_*), strain at break (*A_t_*), and flexural strength (*σ_max_*) were determined.

The hardness was measured based on the Shore D method using an analog hardness tester with the test stand 7206/H04 from Zwick (Ulm, Germany) at standard temperature (23 ± 2 °C). The result was obtained after 15 s. The procedure was followed the EN ISO 868:2003 standard [54]. The final result was the arithmetic averaging of five individual measurements.

#### 2.4.6. FT-IR Spectroscopy Analysis

The characteristic bands for the functional groups of the samples were determined by using the ATR/FT-IR technique. The spectra were measured on a Bruker TENSOR 27 spectrometer (Ettlingen, Germany) in the frequency range from 600 to 4000 cm^−1^ with a resolution of 4 cm^−1^ at 32 scans per sample. The analysis was also preceded by the background spectrum measurements.

## 3. Results and Discussion

Changes are relatively quickly noticeable in the case of thermoplastic polymers while the cross-linked polymers exhibit resistance to destructive factors. Our research aims to answer the question of how the destruction of composites based on cross-linked resins proceeds.

Post-cured samples of the UPR and VER resins and their composites with lignin were studied. Post-curing increased crosslinking degree of the resins, yielding a more rigid materials less susceptible to premature failure [55].

**Figure 1:**
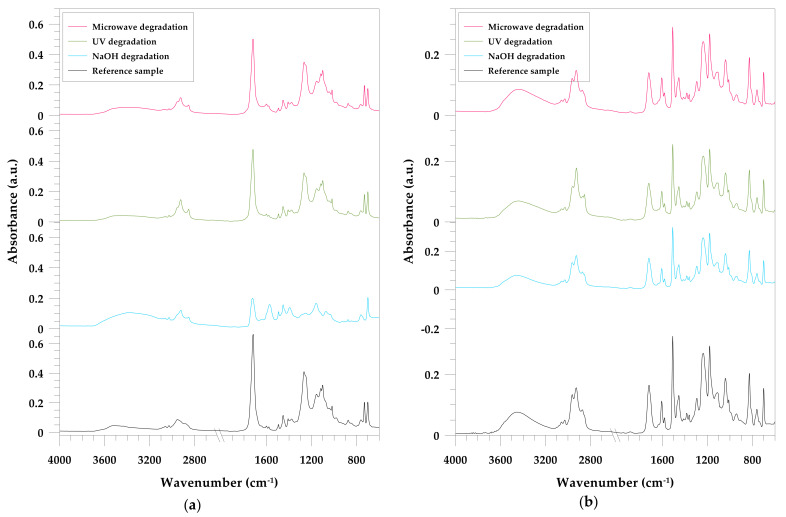
ATR/FT-IR spectra of the: (**a**) pure UPR; (**b**) pure VER.

The results of the chemical structure studies of the initial samples are shown in Figure 1. The ATR/FT-IR spectra of the UPR and VER resins are different. The UPR spectrum was described earlier [56]. It contains high intensity peaks at 1719 and 1260 cm^−1^. A double band at about 700 cm^−1^, representing styrene ring (aromatic –CH ring), is characteristic.

The VER spectrum is dominated by the characteristic absorption bands corresponding to the aliphatic hydrocarbon backbone (symmetric and antisymmetric stretching of the –CH_2_ and –CH_3_ groups at 2963, 2927, 2872 and 2851 cm^−1^, with the corresponding bond at around 1455 and 1362 cm^−1^) and the presence of aromatic rings—aromatic CH stretching vibrations ranging from 3100 to 3000 cm^−1^ (3059 and 3028). The broad band around 3450 cm^−1^ corresponds to the stretching of the associated OH groups.

There are also other bands for aromatic ring stretching at 1607 and 1582 cm^−1^ and out-of-plane bending vibrations of para-bisubstituted and monosubstituted rings at 828 and 701 cm^−1^, respectively, although other groups can be identified. The peaks at 1719 and 1181 cm^−1^ were assigned to stretching of the carbonyl C=O groups and stretching of the C–CO–O fragment from esters, respectively. The vibration stretching of the –COC– groups at 1238 cm^−1^ which is typical for ethers, was also observed.

The VER resin studied is identified as a bisphenol-A (BPA)-based epoxy vinyl ester resin.

**Figure 2:**
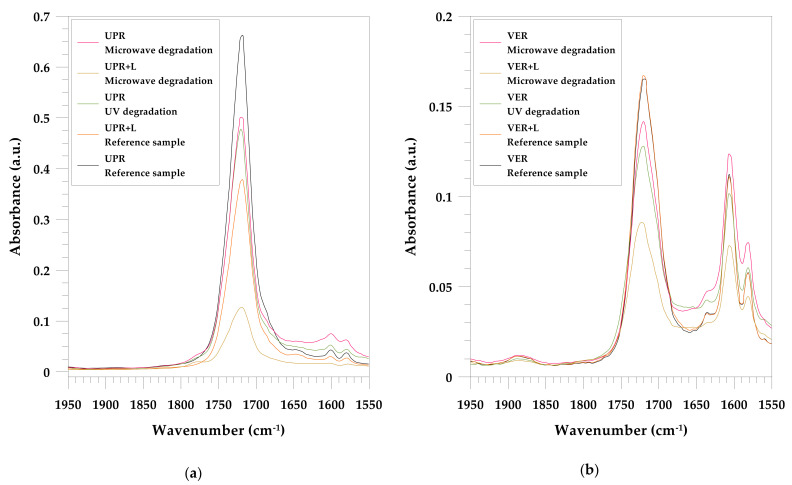
ATR/FT-IR spectra of the carbonyl vibrations’ region for: (**a**) UPR; (**b**) VER.

The Figure 1 and Figure 2 also show the changes in the spectra of the resins after microwave treatment, accelerated UV aging, and immersion in NaOH solution. The spectroscopic analysis of the UPR composites was discussed earlier [56]. In previous studies, a concentrated NaOH solution was used and now immersion tests are performed for a 1% solution.

The evolution in the two major wavenumber regions: 3400–3000 cm^−1^ (hydroxylated groups) and 1900–1600 cm^−1^ (carbonyl vibrations) reveals the formation of the oxidation products.

The degradation of the unsaturated polyester and vinyl ester resins can be investigated by the ester carbonyl (C=O, about 1720 cm^−1^), ester stretching vibration (C-O, about 1230–1260 cm^−1^) and aromatic ring (C–H, 700 cm^−1^). Especially due to the immersion test, the latter does not change during aging because styrene cannot hydrolyzed [57].

In the case of pure UPR (Figure 1a) the spectrum of the material treated with NaOH deserves attention. A decrease in the intensity of absorption bands corresponding to the vibrations of the carbonyl and ester groups was noticed. There is also a more intense band corresponding to the vibrations of hydroxyl groups for alcohols and carboxylic acids. This result suggests that the degradation action based on the hydrolytic breakage of the ester bond occurred. Similar observations were made by Vasco et al. [58] as well as us [59].

During the aging test, UV radiation promotes photo-oxidative, thermo-oxidative and photolytic reactions [60]. Earlier we mentioned that the tested resins turned yellow as a result of aging. This phenomenon is an undesired consequence of photoaging and photooxidation of polystyrene sequences [61]. Changes in their structure were confirmed by the spectra (Figure 1 and Figure 2). The oxidation products, such as mixture of carboxylic acids and ketones were appeared as a result of the photo-oxidative degradation of polyester [62]. This can be ascribed to carbonyl degradation as a result of disruption of the parent polymer chain by the Norrish type II mechanism [63].

According to the FT-IR spectra of the pure VER (Figure 1b), it can be seen that this material is very durable and resistant to various factors. No changes in the spectra were observed. Neither the immersion, nor UV exposure, and even the microwaves did not significantly affect its chemical structure. The aging causes only decreasing of the band intensities of the carbonyl C=O and C–O–C of the ester groups. Similar observations were made by Alia et al. [64].

Michaille et al. observed that photodegraded UPR resin can undergo crosslinking [65]. After degradation, there was no band observed at 1645 cm^−1^ corresponding to the unsaturated bonds. Therefore, it can be concluded that the material was crosslinked also due to the thermal effect during microwave treatment. For resin and composites based on VER, this phenomenon also occurs, but is not so noticeable.

Anhydride formation after degradation was clearly observed only on the UPR spectra especially for microwave-treated lignin composite (1860 and 1780 cm^−1^).

According to FT-IR spectra of both resins and their lignin composite (Figure 2a,b) one can see that after degradation only the carbonyl band (1720 cm^−1^) was broadened and shifted.

**Figure 3:**
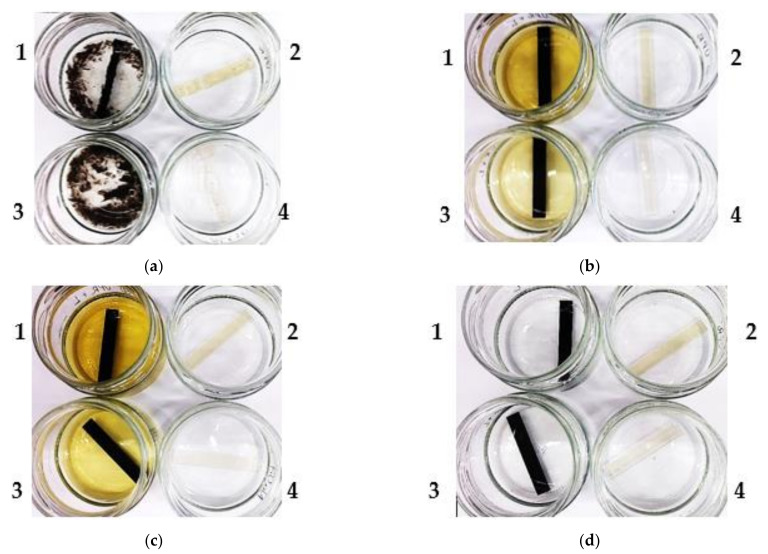
Images of the UPR and VER lignin-based composites during the immersion test in: (**a**) acetone; (**b**) sodium carbonate; (**c**) sodium hydroxide; (**d**) hydrochloric acid. 1—UPR + L; 2—UPR; 3—VER + L; 4—VER.

The changes occurring in the resins and their composites with lignin during the immersion test are presented in Figure 3. During the test, many changes were observed. They appeared not only in the materials themselves but also in some of the liquid chemicals in which they were immersed. A significant influence of immersion on the behavior of UPR and VER composites was observed for acetone. In the case of pure UP resin, after one day of immersion, severe shrinkage effect occurred which caused the cracking of the specimen. The same was observed for the VER resin but the process took some days. In the successive stages the sample underwent crushing into smaller pieces. Due to the addition of kraft lignin, such a strong shrinkage was not observed, but the process of partial delamination and exfoliation took place (Figure 3a). Similarly to our previous study, for both resins and their composites no significant changes were observed in hydrochloric acid (Figure 3d). Only some samples immersed in water and hydrochloric acid were lighter in color, suggesting bleaching.

Some color changes of liquid chemicals were observed for the NaOH and Na_2_CO_3_ solutions (Figure 3b,c). Yellowish or orange-yellowish solutions included leaching of the lignin-biofiller. A more intense color was found in sodium hydroxide, especially for the UPR-based samples. This suggests that the VER composite is more chemically resistant compared to the UPR composite. The superior chemical resistance of vinyl ester resins results from the lack of ester bonds in the epoxy backbone in those sites where the polymer units are linked with phenyl ether bonds. The latter are significantly more resistant to degradation in many chemical environments, especially in an alkaline of high pH. The ester linkages in the VER are only present at the end of the chain molecules, which minimizes their number that can be chemically attacked [22].

Table 2 shows the results of the thermal analysis of pure resins and their composites with lignin. The earlier studies for the UPR indicate two major degradation steps with the maximum decomposition temperature at 394 °C and 519 °C [59]. Its initial degradation temperature (IDT) is equal to 165 °C (167 °C in the earlier determination). The vinyl ester resin is more thermally resistant. Its initial degradation starts at 175 °C whereas maximal degradation takes place at 427 and 518 °C. The addition of lignin causes a significant decrease of IDT for both resin composites, in agreement with the literature [66]. On the other hand, the presence of lignin in both composites is responsible for the existence of residual mass even after heating to 1000 °C. Under these conditions, the process of lignin carbonization began. There is no residual mass for pure resins.

Thermal decomposition of lignin also influences the degradation of composites. In the case of UPR composite, the weight change decreased in the first decomposition area from 85.18 to 73.49%, and in the second one increased from 14.14 to 19.13%. For the VER composites these values are 73.00 and 74.87% in the first step and 26.88 and 15.91% in the second step, respectively.

**Figure 4:**
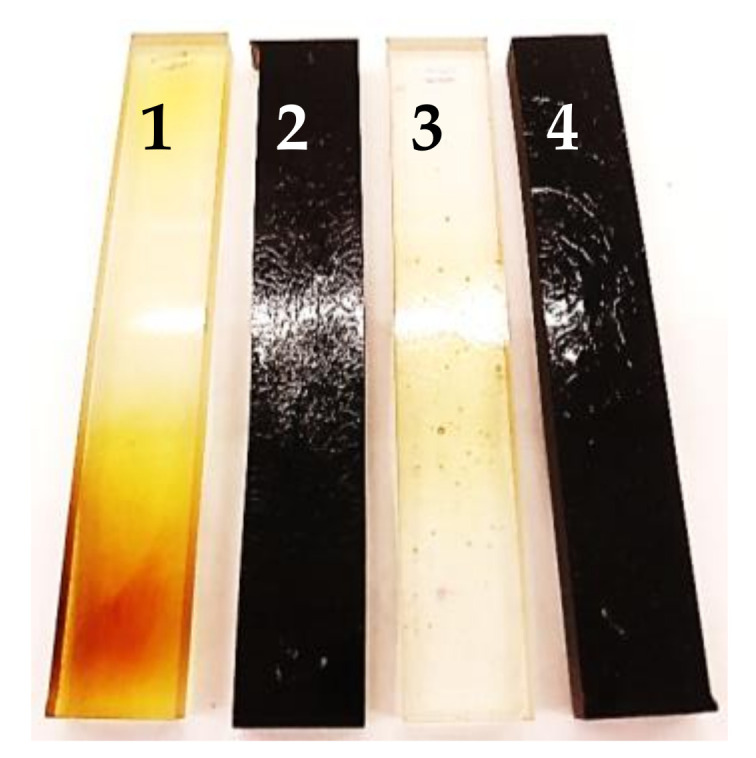
Images of the UPR and VER lignin-based composites after 80 min of microwave irradiation. 1—UPR; 2—UPR + L; 3—VER; 4—VER + L.

**Figure 5:**
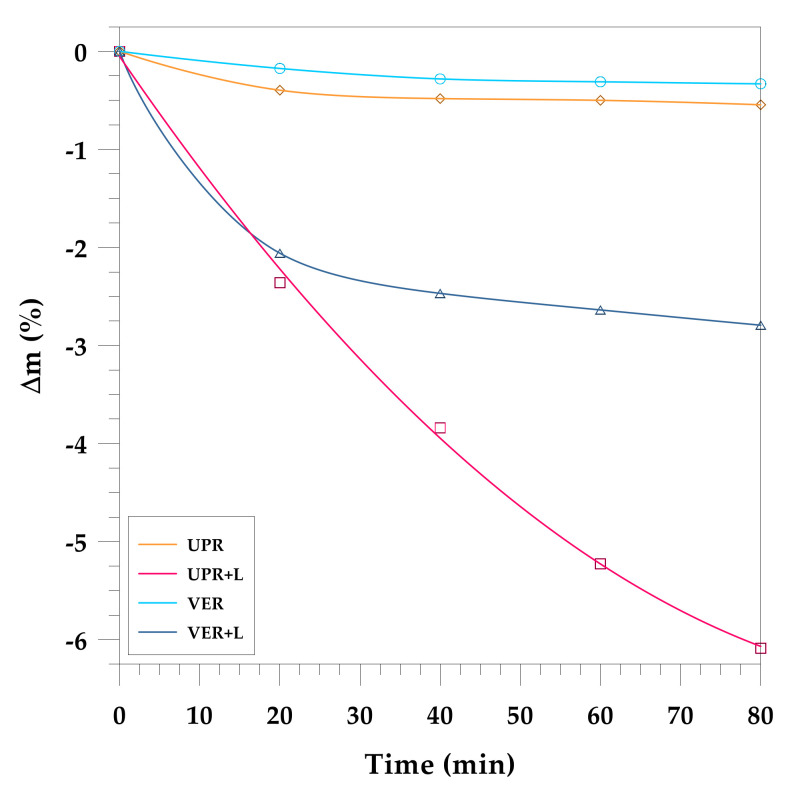
Mass change of the UPR and VER lignin-based composites during microwave irradiation.

Interesting results were obtained on the degradation of resins and their composites from the microwave studies. The studied samples were placed in the microwave reactor on the ceramic pedestals subjected to a microwave power of 1000 W. The samples were examined after 20, 40, 60 and 80 min of irradiation. The obtained results are presented in Figure 4 and Figure 5. Figure 4 shows the photos of the sample whereas Figure 5 shows the relationship between the time of irradiation and the mass change. From these data one can see that microwaves cause a very small weight loss in VER and a slightly greater loss in UPR. However, for the composites it is significantly larger. After 40 min of microwave operation for the composite with VER it was about 2.5% and for the composite with UPR it reached 4%. After the next period of microwave treatment, the mass of VER composite did not change significantly (2.8%) compared to the UPR where the weight loss exceeded 6%. It should be assumed that mainly the lignin was degraded and not the resin matrix. The question is how long should microwaves be used to reduce the mass by 10%.

As a result of exposure into microwaves, significant yellowing and even browning for UPR were observed, while for VER slightly noticeable yellowing appeared. The situation changes dramatically for the composites with lignin. Due to their color, it was difficult to see a change. The only significant change was noticed in the surface appearance of the samples. In the case of the UPR-based composite, the surface was cracked and resembled a mosaic structure or patterns. In the subsequent stages of microwave treatment, the VER-lignin composite showed only sporadic cracks. Thus, it can be stated that the mass for the pure resins does not change significantly with the time of microwave treatment. It can be assumed that for the composites, the filler is responsible for these changes. It was found out that composites with lignin became much softer after the irradiation with microwaves and their simultaneous heating and they became very prone to faster degradation. The pure resins did not show such noticeable changes in their stiffness. Each material hardened over time as it cooled but its hardness varied.

There is some discussion about the precise influence of microwaves on the thermosetting polymer composites. Some researchers suggest that microwaves increase the curing reaction through volumetric heating [67,68]. Our studies of the samples’ properties confirm this observation; see hardness values presented in Table 3.

The greatest noticeable changes in hardness are observed for the microwave-assisted degradation. The materials based on pure resins are subject to secondary curing [69], which increases their value by 0.6 ShD. The situation change dramatically in case of their composites where the hardness decreases, which was possible to predict from their surface cracking. For the UPR-based composite, this change (4.4 ShD) is greater than for the VER + L material (1.5 ShD). Moreover, for UPR + L and VER + L samples treated with UV-light, a decrease of hardness in comparison to pure resins was observed. Phenol groups present in the structure of kraft lignin absorb UV radiation and cause less crosslinking [5].

**Figure 6:**
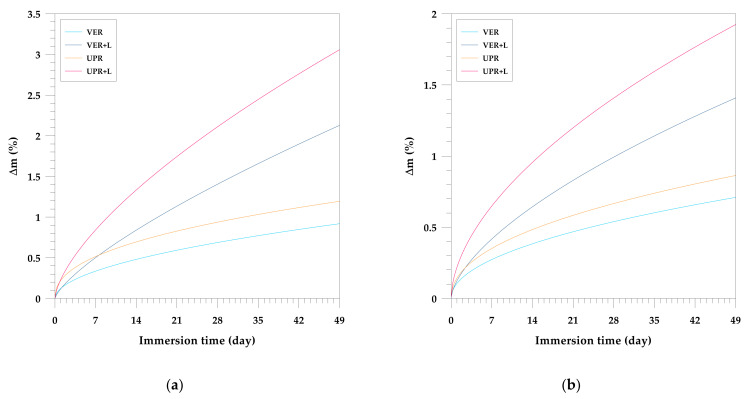

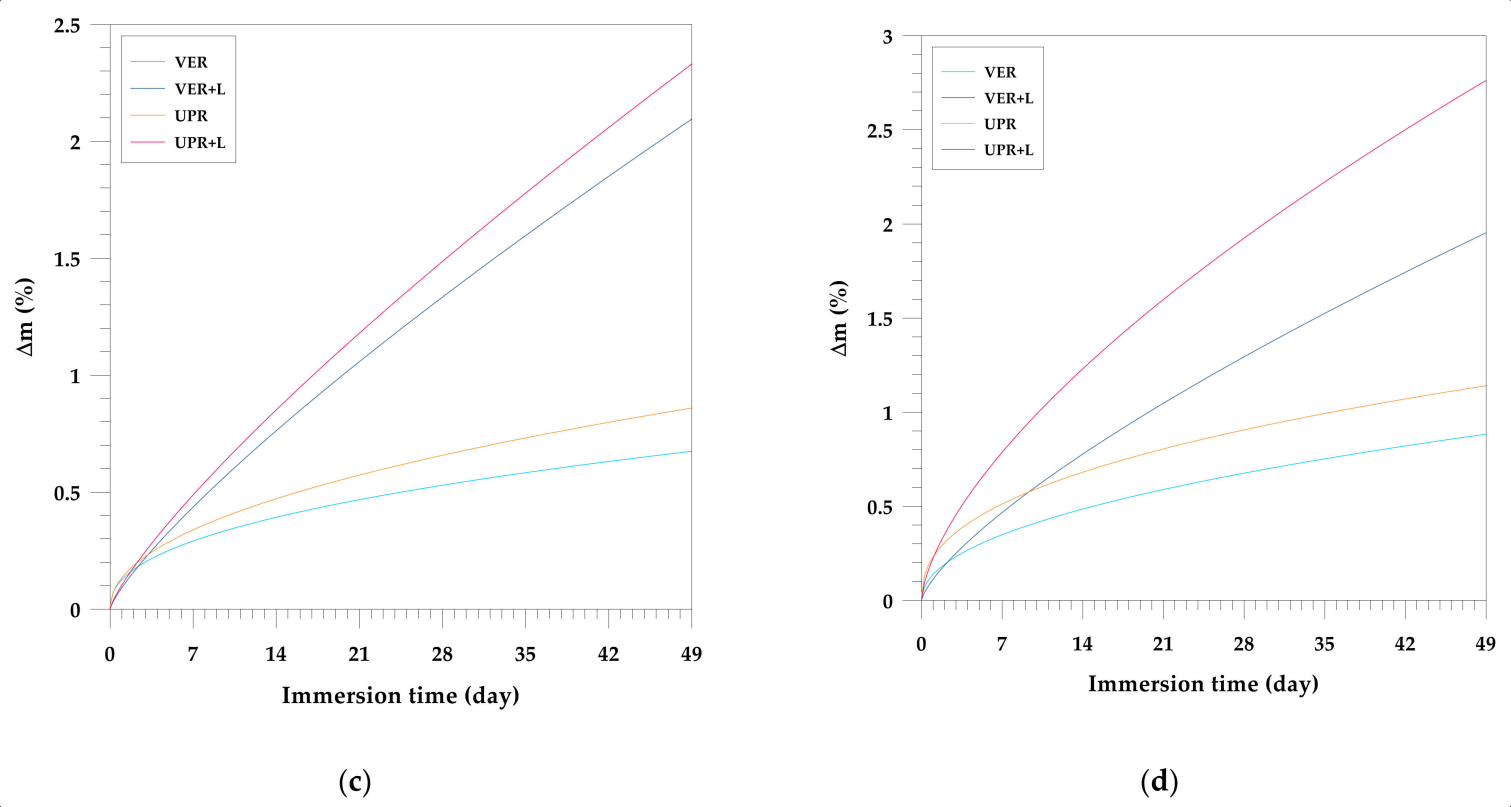
Effect of the chemical resistance of the UPR and VER composites with kraft lignin during immersion test in: (**a**) distilled water; (**b**) 10% HCl, (**c**) 1% NaOH, (**d**) 2% Na_2_CO_3_.

Figure 6 shows the relationships between the mass of the studied composites and their immersion time. It shows exemplary curves obtained in water, HCl, NaOH and Na_2_CO_3_ during the 49-day test. The order of the curves is always the same. The smallest mass changes after the immersion were shown by the VER resin, and slightly larger ones by the UPR resin. The UPR composites with lignin exhibited the largest weight gain. Generally, all samples are characterized by a similar behavior in the aqueous solutions. The greatest weight gain can be observed in water. VER composite absorbs less water than the polyester one. Similar observations were made by Boinard et al. [70]. It is important that the change in the mass of the VER resin after 49 days of immersion does not reach 1%. For the UPR/lignin composites in all environments, the greatest increase in mass is observed. The water absorption process lasted continuously for 49 days.

The lower water sorption is due to the postcuring process in the crosslinked resin, which leads to changes that strengthen the chain organization. This phenomenon prevents the penetration of moisture through water diffusion through the polymer network [71].

The mechanism of lignin degradation is initiated by the attack of H atoms bound to the phenolic OH groups by the hydroxide ions of NaOH. The (OH^−^) ion from an alkaline solution will break the bonds from the basic structure of lignin, while the (Na^+^) ion will bind with lignin to form sodium phenolate. The phenolic hydroxyl group of the lignin is ionized to form a salt. As a phenolic salt, it dissolves easily in water [72,73].

Thermomechanical properties of the studied resins and their composites before and after different degradation treatment are presented in Table 4.

These data show that UPR was characterized by a lower glass transition temperature (129.3 °C vs. 133.3 °C) and a much smaller damping factor (0.48 vs. 0.77) compared to that for VER. The addition of the biofiller (lignin) to the resins in both cases decreased the *T_g_* and damping factor values. Interesting trends can be observed when analyzing the FWHM values. It is intriguing that the values of this parameter for the composites with lignin were smaller than for the pure polymer matrices. Moreover, both pure VER and its composite were characterized by a smaller FWHM value, which indicates its greater homogeneity compared to UPR.

The effect of UV radiation and microwave in the case of UPR and its composite resulted in an increase in the *T_g_* and damping factor values. This suggests that UV radiation and microwaves have additionally “hardened” these materials. For samples immersed in H_2_O and NaOH, a decrease of the values of *T_g_* and damping factor are observed. For VER + L, a significant increase in *T_g_* caused by the action of microwaves is visible, while for VER and its composite, in most cases, the action of physical factors (UV and microwave) or chemical factors (water and NaOH) causes a decrease in the *T_g_* value.

When analyzing the values of the storage modulus, it is difficult to express clear relationships. As can be seen, the *E’* (20 °C) value of the pure UPR resin was higher before than after degradation. The inverse relationship was observed for the VER resin. The addition of lignin to the resins caused an increase in the *E’* (20 °C) value (for VER) or its slight decrease (for UPR). The values of the storage modulus of resins in the elastic state E’ (180 °C) were smaller than in the case of their composites. This is due to the fact that the filler strengthened the composite, and it was visible when the composite was in an elastic state. The degradation process of composites resulted in a reduction in the value of the storage modulus in the elastic state.

Natural fillers improve the mechanical properties of composites, and consequently enhance their resistance to the external mechanical stress. The mechanical forces in the environment are rather minor and usually act as abrasion and erosion agents in combination with other aggressive factors, such as water, dissolved oxygen and salts or UV radiation. On the other hand, mechanical forces are much stronger in processed reprocessing, causing changes in the structure of the materials properties with relatively high temperatures [43].

**Figure 7:**
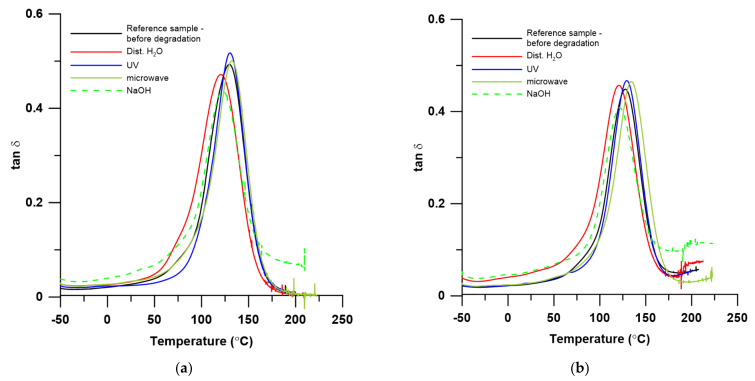

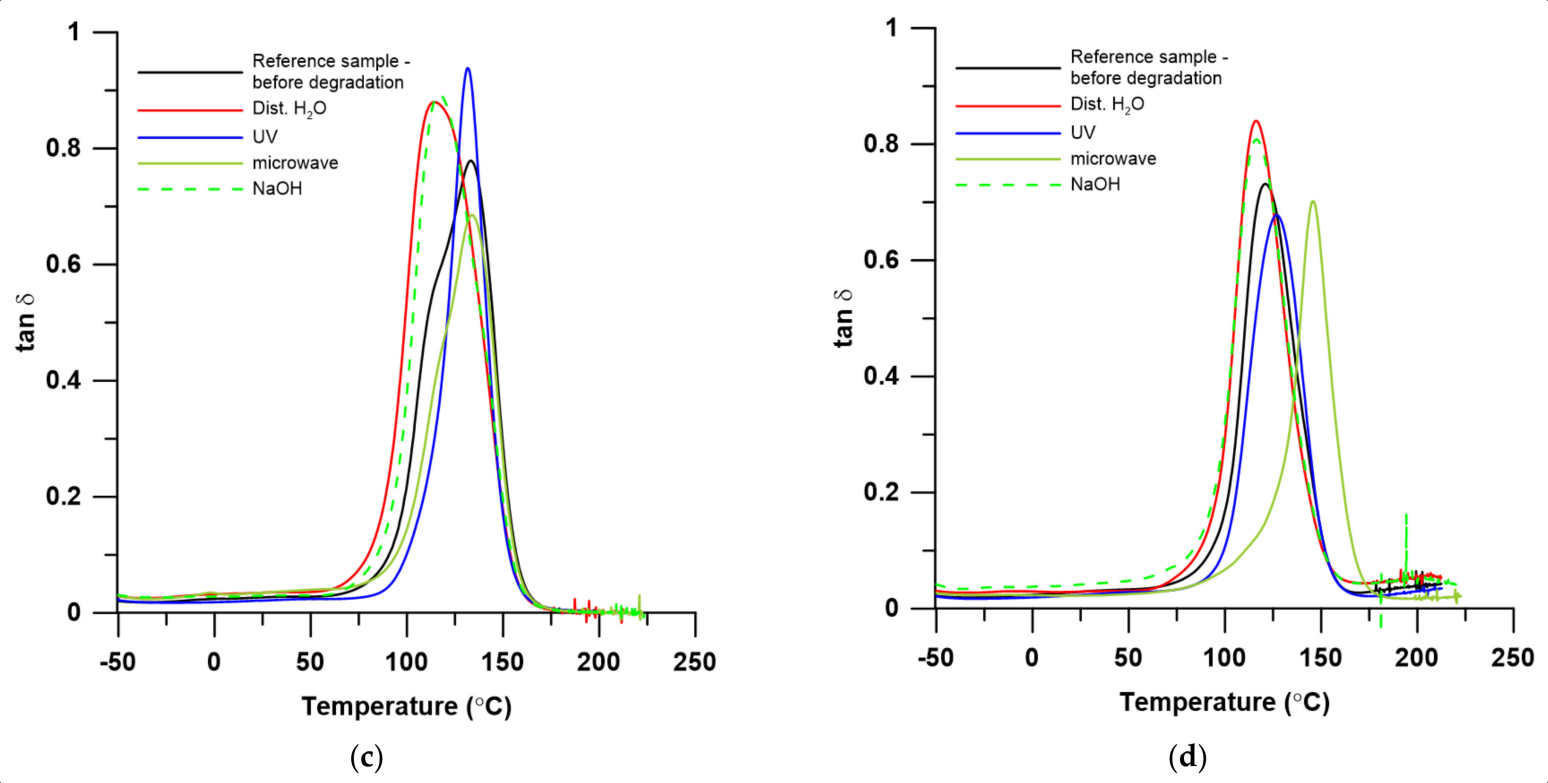
The temperature-dependent graph of damping factor (*tan δ*) before and after degradation: (**a**) pure UPR; (**b**) UPR + L; (**c**) pure VER; (**d**) VER + L.

The results summarizing the research are presented in Figure 7.

The heterogeneity of the sample is reflected on the width of the *tan δ* peak. The Full Width at Half Maximum values for the composites of both resins are narrower comparing to those of the pure resins. A narrow peak indicates their homogeneous structure. It is especially visible for composite samples after exposure to microwaves and UV irradiation in the aging chamber.

The damping values (*tan δ_max_*) of the UPR resin and composites do not change significantly, but for the samples immersed in the NaOH environment, they clearly decreased. For the VER resin, immersion in water and NaOH caused a decrease of *tan δ_max_*, but UV or microwave treatment did not change its glass transition temperature. In the case of the VER composite, the significant influence of microwave radiation is visible. Compared to the starting composite, there is an increase in glass transition temperature. The UV treatment also had a slight influence on the increase of the glass transition temperature.

The incorporation of kraft lignin into the vinyl ester resin caused increasing of flexural modulus value from 3.82 to 3.87 GPa (Table 5). The flexural strength and strain at break of a green composite decreased by 78–60 MPa, and 2.01–1.58%, respectively. In the case of the UPR-based material the situation was different. For the UPR + L, an increase of *A_t_* and *σ_max_* from 1.27 to 1.43%, and from 47 to 51 MPa, respectively, was noticed.

## 4. Conclusions

The degradation of the UPR and VER resins and their composites with lignin was studied.

Both pure resins were not fully cured, so their curing was completed during the accelerated aging test or microwave treatment.

Compared to the UPRs, the VER proved to be more resistant to water degradation. Due to low compactness of the polymeric network and the greater hydrophilicity, the latter resin absorbed a higher amount of water.

The polystyrene chains leave a high amount of free volume between themselves during the crosslinking reaction with the polyester chains, so this aspect reduces the bulk compactness. Their greater hydrophilicity is caused by the penetration of water molecules into its polymer network, which enables an easier degradation process. Moreover, the ester groups present in the UPRs are responsible for the degradation.

In the case of the vinyl ester resin, water detaches the surface layers of material without penetrating into the polymeric network.

The composites are less resistant to degradation. Microwaves reduce the mass as a result of the pyrolysis of the biofiller. In the case of thermal treatment, a charred lignin residue is formed and not a resin residue.

The composites are less resistant to degradation. Microwaves reduce the mass as a result of the pyrolysis of the biofiller. In the case of thermal treatment, a charred lignin residue is formed and not a resin residue. The degradation process of composites resulted in a decrease in the value of storage modulus in the elastic state. Their hardness has also decreased.

The obtained results indicate that both resins and their composites with kraft lignin show high resistance to destructive factors, while the VER resin is more resistant. The exception is acetone, in which the biofiller is rinsed first, and then the resin breaks into pieces.

## Figures and Tables

**Table 1:** Formulation of UPR and VER composites.

Sample	Composition (Weight Proportion)		
	UPR ^1^ (wt.%)	VER ^2^ (wt.%)	L ^3^ (wt.%)
pure UPR	100	-	-
UPR + L	90	-	10
pure VER	-	100	-
VER + L	-	90	10

^1^ UPR—unsaturated polyester resin; ^2^ VER—vinyl ester resin; ^3^ L—kraft lignin.

**Table 2:** Thermogravimetric analysis data for the UPR and VER composites with kraft lignin.

**Sample**	$T_{5\%}{\;}^{1}\;({}^{\circ}C)$	$T_{10\%}{\;}^{2}\;({}^{\circ}C)$	$T_{50\%}{\;}^{3}\;({}^{\circ}C)$	$T_{max}{\;}^{4}\;({}^{\circ}C)$	$MC{\;}^{5}\;(\%]$	$RM{\;}^{6}\;(\%)$
pure UPR	299	336	392	394; 519	−85.18; −14.14	-
UPR + L	294	332	390	392; 478	−73.49; −19.13	2.80
pure VER	341	365	422	427; 518	−73.00; −26,88	-
VER + L	312	357	416	413; 483	−74.87; −15.91	2.65

^1^ Temperature of 5% mass loss; ^2^ Temperature of 10% mass loss; ^3^ Temperature of 50% mass loss; ^4^ Maximum decomposition temperature; ^5^ Mass Change; ^6^ Residual Mass.

**Table 3:** Shore hardness of the UPR and VER composites with kraft lignin before and after degradation.

Sample	Shore Hardness(ShD)			
	Before Degradation	Microwave Treatment	UV Treatment	NaOH Treatment
pure UPR	83.0	83.6	83.4	83.4
UPR + L	82.6	78.2	82.2	82.8
pure VER	82.8	83.4	83.4	83.8
VER + L	82.2	80.7	82.0	82.8

**Table 4:** Thermomechanical data for the UPR and VER composites with kraft lignin before and after degradation.

**Sample**	**Type of Degradation**	$E^{\prime }$ **^1^**				$T_{g}$ **(°C) ^2^**		$tan\;\delta_{max}$ **^3^**		FWHM **(°C) ^4^**	
		$E^{\prime }$ **(20 °C) (GPa)**		$E^{\prime }$ **(180 °C) (MPa)**		**From tan δ**					
		**Before**	**After**	**Before**	**After**	**Before**	**After**	**Before**	**After**	**Before**	**After**
UPR	Dist. H_2_O	3.09	2.70	22.05	21.85	129.3	120.5	0.48	0.45	44.23	47.99
	UV		3.03		22.48		130.6		0.50		38.37
	microwave		3.02		19.97		132.3		0.49		39.98
	NaOH		2.13		26.95		122.4		0.38		42.23
UPR + L	Dist. H_2_O	3.04	2.34	36.09	21.80	127.7	120.7	0.41	0.41	38.13	41.45
	UV		3.16		34.34		129.2		0.43		35.98
	microwave		2.35		25.74		134.4		0.44		38.30
	NaOH		2.16		28.80		121.3		0.33		36.89
VER	Dist. H_2_O	2.82	2.95	13.23	13.81	133.3	114.9	0.77	0.86	41.20	43.02
	UV		3.13		11.79		131.7		0.91		22.40
	microwave		2.87		12.48		134.0		0.67		35.67
	NaOH		2.92		13.53		117.3		0.87		39.28
VER + L	Dist. H_2_O	3.00	2.86	19.56	12.28	121.2	116.2	0.70	0.80	30.72	29.51
	UV		3.15		18.06		126.9		0.65		30.82
	microwave		2.31		17.23		145.9		0.67		21.46
	NaOH		2.23		12.54		116.6		0.76		31.09

^1^ Storage Modulus, Glassy and Rubbery; ^2^ Glass-Transition Temperature; ^3^ Mechanical Loss Factor; ^4^ Full Width at Half Maximum.

**Table 5:** Mechanical data for the UPR and VER composites with kraft lignin before degradation.

Sample	*E_Mod_* (GPa) ^1^	*A_t_* (%) ^2^	*σ_max_* (MPa) ^3^
UPR	3.69 ± 0.02	1.27 ± 0.04	46.86 ± 1.23
UPR + L	3.68 ± 0.01	1.43 ± 0.07	51.41 ± 0.58
VER	3.82 ± 0.03	2.01 ± 0.05	77.70 ± 2.54
VER + L	3.87 ± 0.01	1.58 ± 0.01	59.91 ± 0.86

^1^ Flexural modulus; ^2^ Strain at break; ^3^ Flexural strength.

## Data Availability

The data presented in this study are available on request from the corresponding author.
